# The Challenge of the Yuck Factor in Public Acceptance of Engineered Living Materials

**DOI:** 10.1002/gch2.202400384

**Published:** 2025-04-25

**Authors:** Kristen K. Intemann, Hannah R. Lavoie, Kirke D. A. Elsass, Brandon G. Scott, Robin Gerlach

**Affiliations:** ^1^ Center for Science, Technology Ethics & Society Montana State University Bozeman MT 59717 USA; ^2^ Department of Psychology Montana State University Bozeman MT 59717 USA; ^3^ Department of History & Philosophy Montana State University Bozeman MT 59717 USA; ^4^ Center for Biofilm Engineering Montana State University Bozeman MT 59717 USA

**Keywords:** disgust, living materials, social acceptance of materials, yuck factor

## Abstract

Engineered Living Materials (or ELMs) are an emerging class of materials that utilize microorganisms that can either generate their own structure (such as biofilms) or that can be incorporated into synthetic matrices using technologies (such as 3D printing). ELMs can be designed to have multiple functions, such as biosensing, self‐repair, or bioremediation. Such materials have the potential to address a variety of problems related to sustainability, including water security, energy, and health. One major challenge to widescale social acceptance and adoption of these materials is the so‐called yuck factor, or the propensity these materials may have to elicit disgust reactions. This Perspective provides an overview of social science research directed at the yuck factor to identify the drivers and demographics of disgust experiences and to examine how each of these are likely to arise in relation to ELMs. Strategies for overcoming these challenges are also addressed. Finally, areas where future empirical research is needed to better understand disgust toward ELMs, or particular ELM applications, are identified.

## Graphical abstract

This Perspective provides an examination of the drivers of disgust reactions to biotechnologies and how these are likely to arise toward engineered living materials, which incorporate living cells. To promote social acceptance of such materials, disgust reactions can be minimized though the design and development of such materials, science communication, education, and outreach.

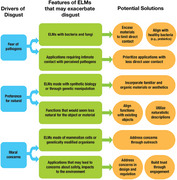

## 1 Introduction

Engineered living materials (or ELMs) are an emerging class of materials that utilize microorganisms that can either generate their own structure (such as biofilms) or that can be incorporated into synthetic matrices using technologies such as 3D printing.^[^ ^1^, ^2^, ^3^, ^4^ ^]^ ELM researchers are utilizing both wild‐type (naturally occurring) and engineered microorganisms including bacteria, fungi, plants, and mammalian cells that can be designed to persist in biologically active materials.^[^ ^1^, ^2^ ^]^ The living cells that form such materials can be designed to have multiple functions, such as biosensing, self‐repair, or bioremediation. For example, hydrogel encapsulated *E. coli* cells can sense heavy metal ions based on the enzymatic activity of the cells, which could provide a low‐cost and highly effective toxicity test for heavy metals in water samples^[^ ^5^ ^]^ and cyanobacteria‐based living materials have been used to remove heavy metals from water.^[^ ^6^ ^]^ Porous ceramics incorporating genetically engineered *E. coli* and wild‐type cyanobacteria have demonstrated the ability to both capture carbon and sense toxic gas.^[^ ^7^ ^]^ Bacteria incorporated into concrete can self‐monitor to identify damage and self‐repair, increasing the durability and longevity of the material and reducing the need for repair maintenance.^[^ ^8^, ^9^ ^]^ Living therapeutics with engineered mammalian cells are being developed for a variety of health applications (such as skin, heart, or bone repair).^[^ ^10^, ^11^ ^]^ Living walls that incorporate plant cells have been found to be effective in removing particulate matter (PM) and volatile organic compounds (VOCs) from air.^[^ ^12^ ^]^ Thus, while there are still technical challenges to overcome, ELMs have exciting potential to address a variety of problems related to sustainability and health.^[^ ^2^, ^3^, ^13^, ^14^ ^]^

Yet while ELMs hold significant promise for social benefits, there are several likely challenges to wide‐scale social acceptance of such technologies, in addition to technical challenges. One significant social challenge is that many novel technologies, particularly biotechnologies, can elicit emotional responses of disgust or what has been referred to as the “yuck factor”.^[^ ^15^, ^16^ ^]^ The yuck factor is an emotional experience of disgust or repulsion that individuals can have toward biotechnological applications, processes, or products. These feelings of disgust may lead people to reject ELMs for personal use or oppose their widespread adoption. This phenomenon has occurred with respect to other biotechnologies, including genetically modified foods,^[^ ^17^, ^18^, ^19^ ^]^ vaccines,^[^ ^20^, ^21^, ^22^ ^]^ and bio nanotechnology.^[^ ^23^, ^24^ ^]^ The yuck factor raises important questions about the extent to which the general public is likely to experience feelings of disgust toward ELMs or particular applications of ELMs, and whether those feelings could either be avoided or addressed during development or through science communication. A review of the literature on the yuck factor shows (1) certain demographics are more likely to experience feelings of disgust toward biotechnologies and (2) there are at least three distinct drivers or triggers of disgust that are thought to be relevant to ELMs: A desire to avoid pathogens, a preference for things to be natural, and conflict with moral or religious views. We examine how each of these drivers is likely to arise in relation to ELMs and assess how they might be addressed through ELM design and science communication. Finally, we identify areas where future empirical research is needed to better understand why ELMs, or particular ELM applications, may elicit feelings of disgust.

## 2 What is the Yuck Factor?

The “yuck factor” is the extent to which individuals are inclined to experience feelings of disgust toward biotechnologies, processes, or materials. Disgust, of course, can be experienced in many contexts. Feelings of disgust can arise toward physical objects (such as dirty diapers), entities (e.g., spiders), sensory experiences (e.g., a foul smell or taste), specific settings (e.g., hospital morgues), or even when confronted with certain ideas and symbols that one might find morally repugnant (such as racist symbols or jokes). Disgust has roots in human evolution but is also influenced by social learning, individual perceptions, cognitions, and behavior.^[^ ^25^, ^26^ ^]^ Disgust emotions can be accompanied by strong physical reactions, such as specific facial expressions (e.g., grimacing or scrunching of the nose), galvanic skin responses, lowered blood pressure, and nausea.^[^ ^27^, ^28^ ^]^ Such emotional experiences play a vital role in creating subsequent thoughts, or cognitions which can impact the way we engage with or behave around objects of disgust.^[^ ^29^ ^]^ For example, rejecting or avoiding materials that are perceived to be disgusting. As a result, the presence of an emotional experience of disgust is the catalyst to beliefs about, and behavioral avoidance of, disgusting things.^[^ ^19^, ^30^ ^]^ Once an experience of disgust has occurred, it is exceptionally difficult to counter.^[^ ^30^, ^31^ ^]^ It is therefore important to understand what may trigger those emotional reactions so that the onset of disgust can be avoided or challenged early.

## 3 Who is Likely to Experience Disgust?

Many studies show that the extent to which individuals find something disgusting is influenced by their level of disgust sensitivity.^[^ ^32^, ^33^, ^34^, ^35^, ^36^, ^37^, ^38^ ^]^ Individuals with a higher level of disgust sensitivity experience disgust more easily and intensely compared to those with lower disgust sensitivity. Conversely, individuals with lower disgust sensitivity typically find fewer things disgusting or may have a less intense emotional response to disgusting things.

There is a large body of research establishing that particular demographics have a higher disgust sensitivity in certain contexts. Research has shown that sex is a significant predictor for disgust sensitivity, with women tending to have higher disgust sensitivity than men.^[^ ^32^, ^39^, ^40^, ^41^, ^42^, ^43^, ^44^, ^45^ ^]^ However, there is debate about the cause of this difference. Some studies suggest that disgust sensitivity may be evolutionarily selected for in women for reasons related to parental investment.^[^ ^40^, ^46^, ^47^, ^48^, ^49^, ^50^ ^]^ For instance, women may have a higher disgust sensitivity to things such as incest or potential pathogens because this tendency helps protect their offspring, whose care and wellbeing they are more invested in directly.^[^ ^47^, ^50^, ^51^, ^52^ ^]^ Some studies suggest that differences might be explained by hormonal sex differences.^[^ ^53^, ^54^, ^55^ ^]^ Others have argued that such differences are likely the result of social influences, such as gendered differences in risk‐taking^[^ ^56^ ^]^ or different gendered norms^[^ ^57^ ^]^ (e.g., that women *should* find certain things disgusting or that men should be tough or strong so as not to be disgusted by much). Some have suggested that disgust sensitivity may be tied to other personality traits (such as agreeableness, conscientiousness, and lack of openness to new experiences), which also may be the result of gendered socialization.^[^ ^32^ ^]^

Age, education level, income, and religiosity can also modulate disgust sensitivity in some contexts, though to a lesser extent than gender.^[^ ^44^ ^]^ For example, several studies have found that disgust sensitivity is lower in those who are older.^[^ ^44^, ^58^, ^59^ ^]^ This may be because those who are older have had a greater range of experiences that makes them more open or tolerant.^[^ ^44^ ^]^ Those that have more STEM education or greater knowledge about science tend to have lower disgust sensitivity toward biotechnologies.^[^ ^60^, ^61^, ^62^ ^]^ Those with more knowledge of science may find biotechnologies more familiar, may be more informed about the risks and benefits, or may simply have more trust in scientists and the institutions governing such technology.^[^ ^62^, ^63^, ^64^ ^]^ Those with lower incomes and less education, particularly those living in communities with higher risks for disease and infection, also tend to have higher disgust sensitivity, presumably because they face greater threats.^[^ ^65^, ^66^ ^]^ Similar research showed that individuals with less education and lower income also tended to be less accepting of wastewater reuse and displayed greater discomfort toward the implementation of those technologies.^[^ ^67^, ^68^, ^69^ ^]^ Those who report strong religious beliefs also appear to have higher disgust sensitivity.^[^ ^36^, ^39^, ^44^, ^70^, ^71^ ^]^ This may be due to the fact that several religions emphasize the importance of purity and cleanliness^[^ ^72^, ^73^ ^]^ or because modifications to nature are perceived to be in conflict with some religious beliefs.^[^ ^74^ ^]^

Yet, the difference that these demographic variables had on disgust sensitivity was slight in some contexts and there is mixed evidence about whether such variables influence disgust reactions across multiple domains.^[^ ^44^ ^]^ For example, while those who have lower incomes have higher disgust sensitivity about infection and disease (perhaps because they live in environments with a higher level of pathogens), they tended to have lower disgust sensitivity to food (possibly because those with higher incomes have the option of being pickier or wasting food, while those with fewer resources do not).^[^ ^75^, ^76^ ^]^ Consequently, more research is needed to determine which demographic variables might modulate disgust sensitivity or impact disgust experiences toward biotechnologies or ELMs specifically. Knowing which demographics have a higher disgust sensitivity in relation to ELMs may help inform communication strategies to reduce disgust reactions to ELMs. In the next section we identify different sources of disgust emotions that are particularly relevant to how ELMS may be perceived.

## 4 Potential Sources of “Yuck” in ELMs

Research on the yuck factor reveals that there are several distinct drivers or sources that are likely to trigger feelings of disgust or repulsion. Three of these are particularly relevant to potential reactions toward ELMs: a desire to avoid pathogens, a preference for what is perceived to be natural, and moral repulsion or a perceived conflict with moral or religious views. We consider each of these sources of disgust and why they may arise in relation to ELMs.

### 4.1 Desire to Avoid Pathogens

Evolutionary psychologists and geneticists make the case that cognitions and reactions of disgust emerged as a heritable advantage that helped avoid disease.^[^ ^41^, ^47^, ^77^, ^78^, ^79^, ^80^, ^81^ ^]^ Recorded history includes many specific examples of people experiencing disgust through smell, taste, or vision, suspecting a source of disease, reinforcing this suspicion among one another, and avoiding the suspected source both individually and collectively.^[^ ^82^, ^83^, ^84^, ^85^, ^86^ ^]^ By avoiding things that are perceived to contain pathogens, humans have likely reduced their exposure to infection or illness and increased their odds of surviving.

ELMs are likely to evoke pathogen disgust insofar as they contain things that will be perceived as pathogens (even if they are non‐pathogenic or have been engineered in ways that may make them nonpathogenic). The microbial communities that may be incorporated into ELMs include bacteria, viruses, and fungi, all of which most people associate with infection, diseases, and other health threats. The knowledge that ELMs contain such entities is thus likely to evoke disgust of pathogens and a desire to avoid such materials.

There is also evidence that humans adhere to the intuitive contagion heuristic, which holds that people tend to experience disgust and engage in avoidance even when the contact with a pathogen or contagion is indirect.^[^ ^87^, ^88^ ^]^ For example, studies have shown that a glass of water with an insect will be found disgusting, even after the insect is physically removed.^[^ ^87^, ^88^ ^]^ Moreover, the perception that the water is contaminated (and disgusting) remains even if it is “cleaned” by boiling or filtering the water.^[^ ^87^, ^88^ ^]^ Therefore, the fact that ELMs might contain bacteria, viruses, fungi or anything likely to be perceived as a pathogen may be enough to elicit a disgust response, even if the user does not come into direct contact with those microbes. This disgust is also likely to be transferred to any other substances that have come into contact with the microbes, such as storm water filtered through ELMs to remove toxins or heavy metals.

The desire to avoid pathogens and the principle of contagion may also compound feelings of disgust toward particular functions or applications of ELMs. For example, several studies have shown that the yuck factor is triggered by the reclamation and reuse of wastewater, particularly for drinking.^[^ ^67^, ^68^, ^69^, ^70^, ^89^, ^90^, ^91^ ^]^ ELMs that are designed to capture and bioremediate wastewater may be perceived as “doubly” contaminated – both because the water is coming from wastewater and potential pathogens are being used to do the “cleaning.”

### 4.2 Preference for “Naturalness”

Another core desire of humans is biophilia, or the preference for things that are natural.^[^ ^19^, ^60^, ^92^, ^93^ ^]^ Of course, the perception of what constitutes “natural” or “unnatural” can be rife with prejudices.^[^ ^92^, ^93^, ^94^, ^95^, ^96^ ^]^ Nonetheless, to maintain biophilia, many humans tend to cognitively categorize their surroundings to maintain a sense of division between the natural world and technology.^[^ ^15^, ^97^ ^]^ When this organization is disrupted, effectively blurring the lines between nature and technology, it creates confusion regarding what is natural and unnatural, thwarting our ability to maintain our preferences.^[^ ^15^ ^,^ ^97^ ^]^ Thus, disgust feelings can arise from confusion about whether a particular phenomenon is natural or not. This constitutes an experience of “yuck” as a discomfort that may be distinct from visceral disgust that can be experienced toward pathogens.

Public perception of ELMs may also be influenced by biophilia and subsequent feelings of disgust. Living things, such as microbes or mammalian cells, are part of the natural world. Yet to some people, the idea that living cells might be “engineered” or integrated into building materials may be perceived as “unnatural” or highly technological. When nature and technology cannot be clearly categorized, experiences of disgust can arise, as has happened with genetically modified foods, CRISPR gene editing, and synthetic biology.^[^ ^16^, ^19^, ^99^, ^100^ ^]^ ELMs, as they also integrate natural elements (i.e., living cells) with technical elements (engineering, 3D printing, infrastructure materials), are also likely to be perceived as unnatural “hybrid technologies” that may evoke feelings of repulsion, discomfort or confusion.^[^ ^98^, ^99^, ^100^ ^]^ This cognitive discomfort elicits a dilemma of acceptance because these applications are not only novel and uncategorized but also in conflict with a preference to have the things in our world remain natural.

There is some evidence that the preference for naturalness is also linked with a contagion heuristic. That is, the alteration of something deemed natural tends to diminish its overall perceived naturalness. For example, in one experiment, some were reluctant to drink milk that had been fortified with a vitamin D_3_ supplement and almost half of the participants were unsure whether more such fortified products should be made available.^[^ ^101^ ^]^ Those same participants generally believed vitamin D_3_ to be important and beneficial but expressed a preference for “natural” sources such as sunlight.^[^ ^101^ ^]^ In another experiment, when subjects were informed that minerals had been removed from a water source, the water was perceived to be “less natural”.^[^ ^102^ ^]^ This shift in perceiving once “natural” things as “less natural” or “unnatural” was more pronounced with chemical than physical alterations and also when distinct biological kinds were combined to produce something new.^[^ ^102^ ^]^ For example, several studies show that people have stronger disgust reactions to transgenic modifications, where DNA from one species is inserted into another as opposed to modifications or genome editing where there is insertion, deletion, or modification of same‐species genetic material.^[^ ^103^, ^104^, ^105^ ^]^ Thus, altering a natural substance, even in a way that is taken to be beneficial, appears to dimmish the perception that it is natural, though degree of disgust may depend on the kind of alteration occurring.

### 4.3 Conflict with Moral or Religious Views

Disgust can also arise when we are confronted with objects, actions, or situations that intuitively conflict with our moral or religious values. Moral disgust is the negative feeling of repulsion or repugnance that we feel when we are confronted with a perceived violation of the things we value or believe to be morally right.^[^ ^105^, ^106^ ^]^ For example, one may feel moral disgust at perceived instances of racism or corruption. Moral disgust is often experienced when confronted with biotechnologies, such as human cloning, when they are perceived to conflict with conceptions of human dignity.^[^ ^105^ ^]^ Some individuals also have disgust toward reproductive technologies or procedures that are perceived to conflict with their religious or moral beliefs that the fetus is a person from the moment of conception.^[^ ^107^, ^108^, ^109^, ^110^ ^]^ Unlike biophilia, it does not involve confusion about the mixing of nature and technology, but it can arise in similar contexts if one holds a moral or religious view that anything unnatural is “bad.” Like other sources of disgust, however, moral disgust can elicit a physical response^[^ ^111^, ^112^, ^113^ ^]^ and it is influenced by disgust sensitivity.^[^ ^23^, ^114^, ^115^, ^116^ ^]^

The question for ELMs research is whether there is anything about ELMs or the technologies used to create them that might be perceived to conflict with moral or religious views. Unlike human cloning or reproductive technologies, ELMs research does not involve human genomes and is not likely to be perceived as a threat to human dignity. Yet there may still be concerns about genetic modification different kinds of cells. In general, genetic modification of animals has encountered more resistance than the modification plants, some of which stems from views about the moral status of animals or worldviews that humans are more similar to animals than plants and other living things.^[^ ^117^, ^118^, ^119^ ^]^ Consequently, some may be less comfortable with ELMs that involve mammalian cells as opposed to plant cells or other sorts of microbes. Yet the genetic modification of and use of mammalian cells in ELMs is unlikely to raise the same ethical concerns as genetic modifications to living mammals because cells are not cognizant. However, ELMs that incorporate any genetically modified cells, whether they come from mammals, bacteria, fungi, or plants, may elicit disgust in those who believe that it is morally wrong to “play God” by altering what is natural.^[^ ^120^, ^121^, ^122^, ^123^, ^124^, ^125^ ^]^ Aversion to changing “what was created by God” does arise in the contexts of many biotechnologies, but it is a relatively small population who believes that every technological intervention is bad.^[^ ^126^ ^]^

More significantly, there are large segments of the public that might have ethical concerns about the ways in which ELMs might impact their lives.^[^ ^14^, ^127^ ^]^ Negative emotional reactions to certain kinds of technologies can involve concerns about the potential safety or health risks,^[^ ^69^, ^90^, ^91^ ^]^ worries about access or cost,^[^ ^91^, ^128^ ^]^ or a lack of trust toward the institutions (profit‐seeking industry, policymakers, or agencies) tasked with regulating them.^[^ ^129^, ^130^ ^]^ These concerns are likely to arise in the case of ELMs as well.^[^ ^14^, ^128^ ^]^

## 5 Minimizing Disgust in ELMs Development, Communication, and Education

Despite these potential sources of disgust, there are promising strategies that might be used to address or minimize such reactions. Negative emotional reactions, including disgust, can be at least partially rationally based and this means that some concerns might be addressed to avoid disgust reactions.^[^ ^131^ ^]^ Given the varied potential sources of disgust, countering these reactions will require a combination of different strategies.

### 5.1 Preventing Disgust in ELMs Design and Development

ELMs might be designed in ways that give rise to more positive experiences. There is some evidence that disgust about pathogens can be mitigated by other sensory or cognitive experiences. Empirical studies have shown a strong connection between feelings of disgust and the olfactory cortex, and the evidence suggests a bidirectional relationship.^[^ ^132^, ^133^, ^134^ ^]^ This means that not only can smells trigger disgust but that also feelings of disgust prompted by cognition and other senses can engage the olfactory system to reify and interpret the disgust feeling. For example, in the nineteenth century, historical evidence suggests that odors influenced how people assessed the healthiness of their surroundings and determined the social respectability of where they lived.^[^ ^82^, ^85^ ^]^ Pleasurable olfactory sensations can also influence attitudes and cognitions.^[^ ^135^, ^136^ ^]^ Consequently, intentionally designing ELMs to promote certain kinds of sense experiences (such as having a nice smell) might help prevent cognitions or attitudes that lead to disgust. Similarly, positive cognitions can influence olfactory experiences that individuals have; for example, the smell of waste from domesticated animals has evoked less negative sensory experiences among people who associated it with the tastes of meat and milk, the warmth of wool, or the range of physical comforts that could result from marketing the animals.^[^ ^82^ ^]^ This suggests that having positive cognitions about ELMs, or experiencing them as materials that provide particular benefits, may also decrease disgust experiences.^[^ ^137^ ^]^ If ELMs are cognitively associated with specific benefits (such as sustainability or health) this may help influence how they will be experienced.

Utilizing familiar microbes or materials that people already have positive experiences with may also help foster positive associations and minimize the yuck factor. For example, people in certain regions have already embraced bacteria‐containing biofilms in traditional foods, such as kombucha (fermented tea), kefir (fermented milk product), kimchi (fermented cabbage), tempeh (fermented soybean), and nata de coco (jelly snack from fermented coconut water). Some ELM researchers are working on developing ELMs with microbial cellulose that might have these positive associations for those already familiar with these processes in traditional foods.^[^ ^138^, ^139^, ^140^ ^]^ Beginning with materials or processes that are familiar to humans (or to particular communities) will likely elicit a stronger and faster level of social tolerance or acceptance.

Prioritizing the development of certain ELM applications over others may also help avoid disgust from pathogens or biophilia, particularly when they are initially introduced to society. Previous research shows that feelings of disgust are particularly strong in relation to the body and the protection of one's personal space.^[^ ^66^, ^141^, ^142^ ^]^ This suggests that applications of ELMs that involve less intimate or direct contact with users may be less likely to evoke a disgust response. Various studies indicate that the contagion heuristic is minimized when the contact with a contagion is more indirect. For example, studies on wastewater reuse suggest that people did not experience as much disgust when the water was not only purified but also diluted with non‐wastewater.^[^ ^30^, ^130^ ^]^ That is, they were more comfortable with indirect wastewater reuse and less comfortable with direct reuse “from toilet to tap.” Another study had similar findings in which participants who were concerned about using recycled water for indirect home use (irrigation for gardens/crops), were less likely to support more direct and consumption‐focused applications (bathing and drinking).^[^ ^31^ ^]^ Disgust toward nanotechnologies also was found to be more intense when the applications required more intimate interactions between users and nanoparticles or nanobots, such as those absorbed through the skin or injected into the body.^[^ ^90^, ^143^, ^144^, ^145^ ^]^ Researchers might minimize disgust reactions, then, by prioritizing applications that involve less direct or intimate user contact with things that might be perceived to be pathogenic, such as developing ELMs for civil infrastructure outside of the home (such as roads, bridges, highway barriers, parking lots, roofs and sidewalks). More intimate uses of ELMs may eventually be possible, but again this will be easier when the public is more familiar with such materials.

Prioritizing applications that are more likely to be perceived as natural may also provoke less disgust. This might include selecting functions for microbes used in particular materials that will align with the current uses of those materials. For example, ELM countertops with the function to self‐repair might be viewed as “less natural” because those functions are not currently a part of the functions of non‐living countertops. Consumers are used to thinking of countertops as not only non‐living, but in fact a place where microbes should be killed, leading to the kind of confusion that occurs when biophilia is triggered. In addition, because non‐living countertops do not currently involve functions like biosensing or self‐repair, it may make such materials seem even more “unnatural”. Similarly, water or air filters containing microbes capable of removing harmful particulate matter, viruses, or bacteria might be perceived as “more natural” because the capability aligns with the function of traditional filters. It is also possible that ELMs would be perceived to be more natural if microbes were integrated with other materials considered to be more “natural”, such as glass, wood, bone, stone, or cotton. Even if the materials were not themselves natural, ELMs might be designed so as to result in structural forms that appear more natural. For example, bioremediating ELMs for the ocean that appeared to look like coral or having a surface that might look like waves in the water.

Moral disgust might also be minimized or avoided by addressing those key ethical concerns early in the design of ELMs. For example, designing ELMs to be safe and ensuring that any risks are lower than or comparable to current materials may minimize concerns about safety before they arise in the minds of the public. Particular materials used for ELMs may raise different concerns. For example, some bacteria may be dangerous to human health if they were to escape from the materials, and this needs to be accounted for in the design of ELMs. The use of fungi may pose risks to those who have fungal allergies or sensitivities and therefore ensuring that such sensitivities are not triggered (or selecting alternative microbes) will be important.

### 5.2 Rethinking Language and Communication

How researchers describe ELMs and communicate their functions to the public can also influence whether or not disgust reactions are elicited. Consider, for example, the case of probiotic supplements. Probiotic cultures can provoke a fear of pathogens, but some studies have shown that attitudes can shift when people believe that the bacteria involved are “good” and have beneficial health effects.^[^ ^146^, ^147^, ^148^, ^149^ ^]^ Those most likely to use probiotics and believe they were “healthy” were younger, educated, women, and particularly those that valued health.^[^ ^146^, ^149^ ^]^ Therefore, framing ELMs as materials with “good” microbes that can have specific benefits may help prevent a pathogen‐disgust response toward ELMs.

There may also be ways of describing and explaining ELMs or biofilms that are less likely to evoke disgust caused by biophilia or a preference for the natural. For example, talking about beneficial microbes that can help us in various ways may sound more natural than “genetically modified bacteria that have been engineered to have multiple functions”. Even the term “engineered living materials,” may inadvertently put emphasis on the hybrid nature of this technology that is likely to evoke disgust in relation to biophilia. That is, it is a term that emphasizes that such materials are *both* living (natural) *and* engineered (technical). There is some evidence that describing biotechnologies in ways that emphasize their naturalness or relates them to things that are familiar may be helpful in avoiding negative emotional reactions.^[^ ^89^, ^99^ ^]^ For example, the description of “purified water” tended to evoke fewer negative emotional reactions than “treated wastewater,” “recycled water,” or “bioremediation”.^[^ ^84^ ^]^ Describing ELMs or their applications as natural may also help minimize disgust. Indeed, referring to ELMs as “Living Materials” rather than “Engineered Living Materials” may be less likely to trigger disgust related to biophilia. Similarly, ELMs that are used to bioremediate water, for example, might be referred to as “probiotic water purifiers.”

Finally, particular kinds of communication strategies might also proactively counter moral disgust. For example, describing ELMs as “sustainable living materials” might not only emphasize their naturalness, but also their social benefit.

### 5.3 Science Education, Outreach, and Engagement to Minimize Disgust

If we better understand which demographics might have higher disgust sensitivity toward ELMs (or aversion to biotechnologies in general), education and outreach efforts might prioritize those demographics with higher disgust sensitivity or tailor communication to the concerns of particular groups. For example, if women have higher disgust sensitivity toward potential infections or contagions, then education strategies might emphasize the extent to which ELMs are beneficial for the health of their families and their environment. If greater knowledge of science lowers disgust sensitivity toward biotechnologies, then interventions to promote early science education or ELM‐specific education for the general public might help. Better understanding why disgust sensitivity is heightened for specific groups in particular contexts can be helpful for identifying the best ways to address this difference.

Education and engagement efforts that explain not just the science of ELMs, but that emphasize their social or environmental benefits can also minimize disgust reactions. For example, education about the specific benefits of ELMs or demonstrations of how they serve diverse values that align with items people widely care about can prevent the perception that such materials conflict with moral beliefs. Moreover, being transparent about any risks and how those have been addressed can alleviate potential concerns and facilitate trust between experts and the public.^[^ ^19^, ^30^, ^31^, ^67^, ^130^, ^150^ ^]^ Facilitating trust and addressing ethical concerns that may drive moral disgust will require engaging with those who may be users of ELMs throughout the development process. A proactive and continuous approach would ensure that any self‐imposed regulations developed address these concerns and that researchers are responsive to public interests.

## 6 Conclusion

**Figure 1:**
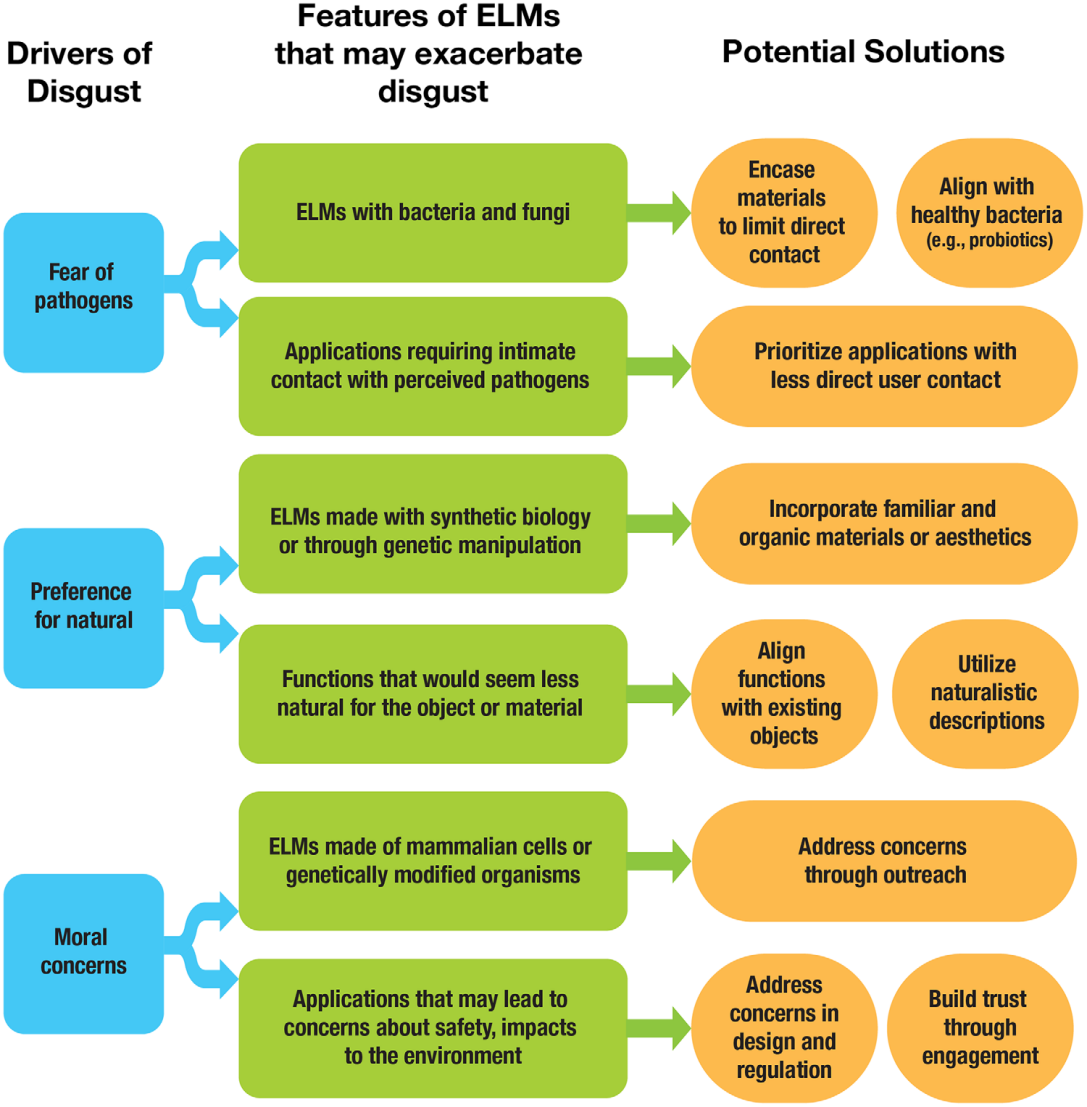
Features of ELMs that may provoke disgust reactions from different sources of disgust and potential solutions.

ELMs have a low level of technology‐readiness, and, as a result, this technology is still largely unfamiliar to the general public. While the emotional reactions specific to ELMs have not yet been studied in depth, previous studies on the yuck factor and disgust toward other biotechnologies suggests that at least three sources of disgust are likely to arise with respect to ELMs, for at least some populations. First, ELMs are partly constituted by components that are likely to be perceived as pathogens (even if they are rendered safe), triggering pathogen disgust. Second, they involve elements that are likely to be perceived as both “natural” and “unnatural”, a fact that may cause some cognitive dissonance given the human tendency toward biophilia. Third, ELMs may trigger moral disgust if their design or use conflicts with particular religious or moral beliefs (such as the conviction that humans should not “mess with” nature or that ELMs may be unsafe). **Figure** **1** (below) illustrates how particular features of ELMs may trigger the three different sources of disgust.

It is not just that ELMs may be comprised of materials capable of triggering disgust, they may also involve functions (such as bioremediation, production of pharmaceuticals, tissue generation, etc.) that are likely to trigger disgust (either from fear of pathogens or the perception that they are “unnatural”). Moreover, because humans tend to operate under a heuristic of contagion, removing or buffering contagions may not be enough to overcome feelings of disgust.

Given that there are different potential sources of disgust (the desire to avoid pathogens, a preference for the natural, and moral convictions) it will be important to distinguish which of these sources is triggering disgust reactions to ELMs in different populations. Depending on the source, there are potentially promising strategies for minimizing disgust through ELM design and regulation, communication about ELMs, and science education and engagement (as Figure 1 shows). Knowing which strategies will be effective, however, requires greater knowledge of the exact concerns stakeholders might have about ELMs or different applications of ELMs.

Additional research is needed to increase our understanding of the yuck factor with respect to ELMs specifically and their different potential applications. Research questions that need to be addressed include:

- Which demographic groups tend to experience disgust toward ELMs specifically?
- Are some types of materials used in ELMs (e.g., bacteria, fungi, plants, or mammalian cells) or processes used to give the matierals structure (e.g. encasing cells in 3D printed hydrogels) more or less likely to provoke disgust reactions?
- What is the source of the disgust when it arises?
- In cases of moral disgust, are there issues that might be addressed (concerns about safety, trust, or equity) through regulation, how ELMs are designed, or through science education?
- Can framing discussions and presentations about ELMs in ways that make clear their natural and non‐pathogenic elements help prevent or alleviate disgust reactions?
- Are remote (more indirect) uses of ELMs less likely to evoke disgust than more intimate uses (that might involve consumption or touching)?

While the properties and uses of ELMs may provoke disgust, it is possible to identify this early in the development process. Understanding the challenges that disgust poses can help ensure that ELM design, communication, and engagement can occur in ways that minimize, rather than exacerbate, disgust reactions.

## Conflict of Interest

The authors declare no conflict of interest.
